# Experimental anti-tumor effect of emodin in suspension – *in situ* hydrogels formed with self-assembling peptide

**DOI:** 10.1080/10717544.2021.1971795

**Published:** 2021-09-02

**Authors:** Weipeng Wei, Jianhua Tang, Lei Hu, Yujie Feng, Hongfang Li, Chengchen Yin, Fushan Tang

**Affiliations:** a Department of Clinical Pharmacy, Key Laboratory of Basic Pharmacology of Guizhou Province and School of Pharmacy, Zunyi Medical University, Zunyi, China; b Key Laboratory of Basic Pharmacology of Ministry of Education and Joint International Research Laboratory of Ethnomedicine of Ministry of Education, Zunyi Medical University, Zunyi, China; c The Key Laboratory of Clinical Pharmacy of Zunyi City, Zunyi Medical University, Zunyi, China; d Cancer Research UK Manchester Institute, The University of Manchester, Manchester, UK

**Keywords:** Self-assembling peptide RADA16-I, *in situ* hydrogels, emodin, cancer therapy, drug delivery system

## Abstract

Lung cancer is a major cause of cancer-related deaths worldwide. Stimulus-sensitive hydrogels, which can be formed by responding to stimuli in the cancer microenvironment, have been widely studied as controlled-release carriers for hydrophobic anticancer drugs. In this study, self-assembling peptide RADA16-I was used to encapsulate the hydrophobic drug emodin (EM) under magnetic stirring to form a colloidal suspension, and the colloidal suspension (RADA16-I-EM) was introduced into environments with physiological pH/ionic strength to form hydrogels *in situ*. The results showed that RADA16-I had good cell compatibility and the RADA16-I-EM *in situ* hydrogels can obviously reduce the toxicity of EM to normal cells. In addition, compared with free EM (in water suspensions without peptide) at equivalent concentrations, RADA16-I-EM *in situ* hydrogels significantly reduced the survival fraction of LLC lung cancer cells, while increased the uptake of EM by the cells, and it also induced apoptosis and cell cycle arrest in the G2/M phase more significantly and reduced the migration, invasion, and clone abilities of the cells *in vitro*. The RADA16-I-EM *in situ* hydrogels also showed better cancer growth inhibition effects in cancer models (mice bearing LLC cells xenograft cancer), which induced cell apoptosis in the cancer tissue and reduced the toxic side effects of EM on normal tissues and organs *in vivo* compared with the free EM. It was revealed that RADA16-I can be exploited as a promising carrier for hydrophobic anticancer drugs and has the potential to improve the administration of anticancer drugs to treat cancer effectively with enhanced chemotherapy.

## 1. Introduction

Recently, cancer has become one of the major malignant diseases causing human death, and its morbidity and mortality are increasing. Possible treatment strategies include chemotherapy, surgery, radiotherapy, immunotherapy, and gene therapy (Wang et al., 2015; Wei et al., 2017; Bykov et al., 2018). However, due to surgical resection of cancers and recurrence, the nonspecific accumulation of radiation or chemotherapeutic drugs in healthy tissues and cells limited their applications in clinical treatment for the lack of targeting, unfavorable side effects, and multidrug (Xiong et al., 2015; Ostrom et al., 2017; Mahvi et al., 2018). At the same time, due to the poor water solubility, rapid degradation in the liver and rapid excretion in the kidneys, most anticancer drugs currently used cannot successfully accumulate at targeting cancer sites, resulting in low efficacy. Except the above, the toxic and side effects caused by addition of excipients or solvents also limit the clinical use of many antitumor drugs (Almeida et al., 2014; Khaliq et al., 2017; Cullen et al., 2020). To overcome these limitations, localized drug delivery systems, such as hydrogels (Pan et al., 2018), micelles (Debele et al., 2017; Xiang et al., 2018), liposomes (Park, 2018; Zhu et al., 2020), nanoparticles (Lin et al., 2017), and electrospun fibers (Ding et al., 2019; Feng et al., 2019) are recently investigated to extend circulation time of the cancer drug in body and release the drug slowly, while reducing the toxicity of the drug to normal tissues and enhancing the therapeutic efficacy.

**Figure 1.**
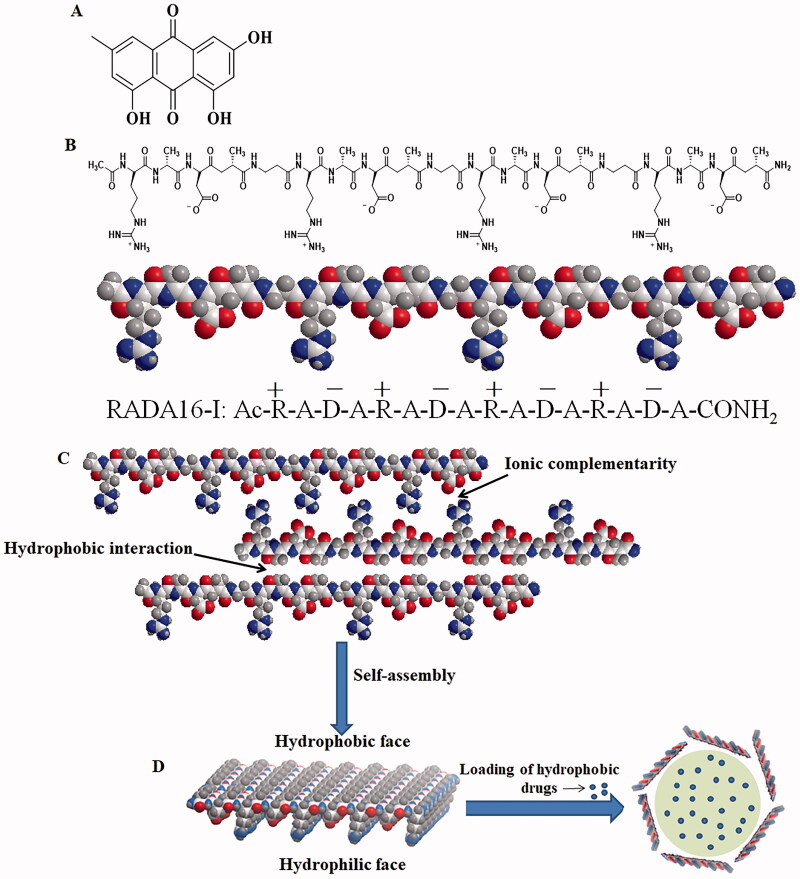
(A) Chemical structure of EM; (B) schematic diagram of charge distribution and three-dimensional molecular model of self-assembling peptide RADA16-I; (C) a scheme of RADA16-I self-assembly through hydrophobic interaction and ionic-complementarity; (D) a proposed model of RADA16-I self-assembly to form nanofiber structures (Liu et al., 2011) and schematic representation of the interaction between hydrophobic drugs and RADA16-I. Oxygen atoms are red, nitrogen atoms are blue, carbon atoms are white, and hydrogen atoms are gray. In this conformation, all of the hydrophobic alanine side chains are aligned on one side, and all of the arginine and aspartic acid side chains are aligned on different sides to produce two different faces: the hydrophobic and the hydrophilic. On the polar side, arginine alternates with aspartic acid. The dimensions are about 5 nm in length, 1.3 nm in height, and 0.8 nm in width.

Emodin (EM) is a natural anthraquinone derivative extracted from the rhizome of rhubarb, polygonum cuspidatum, polygonum multiflorum, and other traditional Chinese medicines (shown in Figure 1(A)). Many studies have shown that EM has anti-inflammatory, bacteriostatic, and immunomodulatory effects (Lee et al., 2010; Lu et al., 2011; Alisi et al., 2012). With the further study of the anticancer activity of EM, the researchers also found that EM can treat lung cancer, breast cancer and other cancers by inhibiting cancer cell proliferation, migration, and promoting its apoptosis (Fu et al., 2007; Li et al., 2013). As EM is practically insoluble in water, easily precipitating to form crystals in water solution, and its characteristic of sudden release in a short time, together with its large side effects, EM has limited clinical use. Even in *in vivo* and *in vitro* experiments, it is inevitable to use some organic solvents such as dimethyl sulfoxide (DMSO), ethanol, or methanol before they can be used. The use of solvents, such as DMSO, which is toxic to the body, limited the administration concentration of EM (Park et al., 2019; Tu et al., 2019). Therefore, EM’s water solubility is crucial to treat cancer, it is necessary to develop a suitable drug delivery system to improve the delivery of EM and other hydrophobic drugs, to improve the efficacy while reducing the side effects of these drugs.

Injectable hydrogels have attracted much interest due to fewer side effects, easy administration, local treatment, and controllable drug release. Some studies have reviewed its application in some fields, such as tissue engineering and antibacterial therapy (Li et al., 2018; Zhang et al., 2019; Qiu et al., 2020). Especially, ‘smart’ hydrogels demonstrate a sol–gel transition responding to external stimuli, such as pH, light, and temperature (Hu et al., 2017; Milcovich et al., 2017; Wei et al., 2020). It has injectable solution state before administration, and it can be transformed into gel state immediately under the stimulation of external conditions (Chu et al., 2013; Morsi et al., 2016; Norouzi et al., 2016). Injectable hydrogels have certain advantages. First, it is usually administered by intra-tumoral and peri-tumoral injection, which helps to increase the concentration of the drug at cancer sites and reduce systemic toxicity. Second, it is simple to make its formulation, which can be prepared without crosslinking agents, organic solvents, and chemical synthesis (Yin et al., 2021). Moreover, it can deliver various therapeutic agents for different models of cancer therapy, such as chemotherapy (He et al., 2015), photo-thermal therapy (Zeng et al., 2019), gene therapy (Saravanan et al., 2019), etc.

Ion complementary self-assembling peptides have attracted more and more researchers' attention as drug carrier materials. As they are synthesized from natural amino acids, they have good biosafety (Eskandari et al., 2017; Qiu et al., 2018; Cong et al., 2020; Tarvirdipour et al., 2020). RADA16-I is the most typical self-assembling peptide in the ion complementary self-assembling peptide family. The hydrophilic amino acids in the structure of RADA16-I include positively charged arginine (Arg, R) and negatively charged aspartic acid (Asp, D). The hydrophobic amino acid is non-polar amino acid alanine (Ala, A) (shown in Figure 1(B)). RADA16-I can be self-assembled into a secondary structure with β-sheets by ion complementarity and hydrophobic interaction in aqueous solution, in which all hydrophobic and hydrophilic amino acids are arranged on different sides, and finally further assembled to form nanofibers (Liu et al., 2011; Shamsi, 2016; Taghavi et al., 2018; Wu et al., 2018) (shown in Figure 1(C)). The hydrophobic regions of its high-level structure can encapsulate the hydrophobic drugs, while the hydrophilic regions can keep the system stable in aqueous solution (Figure 1(D).

**Figure 2.**
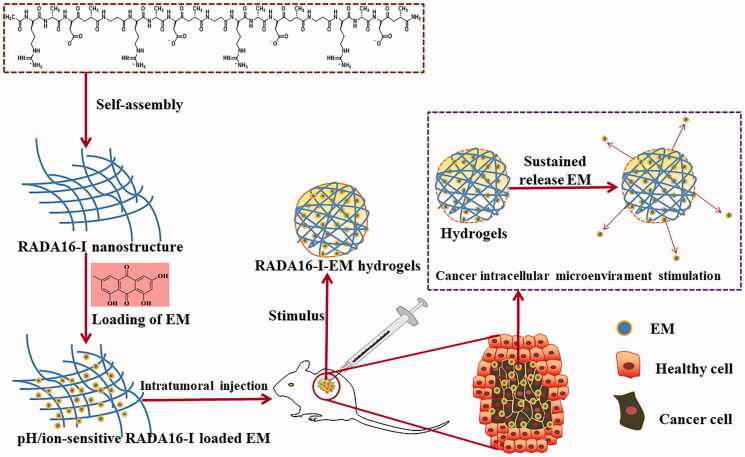
Illustration to develop pH/ion responsive RADA16-I hydrogels loaded with an anti-cancer drug – EM for cancer treatment. The RADA16-I-EM suspension was injected into a cancer bearing mouse model through intra-tumoral injection, while the suspension immediately formed hydrogels *in situ* under the stimulation of the microenvironment in the cancer. Sustained release of EM from the hydrogels inside or beside the cancer exerts inhibitory effects on the cancer cells.

Previous studies have shown that RADA16-I can load hydrophobic anticancer drugs, such as paclitaxel (Liu et al., 2011), 5-fluorouracil (Ashwanikumar et al., 2016), and mangiferin (Meng et al., 2019). It can improve the solubility and stability of anticancer drugs in aqueous solution, so to effectively improve the delivery and release of anticancer drugs. Therefore, based on the previous studies on the construction and sustained and controlled release of RADA16-I-EM suspension-*in situ* hydrogels drug delivery system (Wei et al., 2019, 2021), this study mainly focused on the effects of RADA16-I-emodin hydrogels on lung cancer through the cell experiments *in vitro* and cancer local drug delivery *in vivo* of the constructed RADA16-I-EM *in situ* hydrogels delivery system (Figure 2), and the feasibility of RADA16-I as a hydrophobic drug carrier material was further evaluated.

## 2. Materials and methods

### 2.1. Materials

Emodin (purity ≥ 98%) was purchased from Nantong Feiyu Biotechnology Co., Ltd. (Nantong, China). Self-assembling peptide RADA16-I (1712.77 g/mol, Ac-RADARADARADARADA-CONH2, purity ≥ 98%) was purchased from Shanghai Biotech Bioscience & Technology Co., Ltd. (Shanghai, China). 3-(4,5-Dimethylthiazol-2-yl)-2,5-diphenyltetrazolium bromide (MTT) was purchased from Beijing Solarbio Technology Co., Ltd. (Beijing, China). Trypsin–EDTA solution (0.25%) was purchased from Biosharp Inc. (Shanghai, China). 4′,6-Diamidino-2-phenylindole (DAPI) was purchased from Beijing Pulilai Gene Biotechnology Co., Ltd. (Beijing, China). BD Pharmingen™ cell cycle kit was purchased from BD Biosciences (Shanghai, China) and Annexin V-Alexa Fluor 647/PI apoptosis detection kit was purchased from Beijing Jinpulai Biotechnology Co., Ltd. (Beijing, China). H&E were purchased from Zhuhai Besso Biotechnology Co., Ltd. (Zhuhai, China). TUNEL kit was purchased from the Swiss Roche Group (Shanghai, China). Ki-67 was purchased from Wuxi Aurui Dongyuan Biotechnology Co., Ltd. (Wuxi, China). Cell culture related consumables were all purchased from Corning, Inc. (Corning, NY).

### 2.2. Preparation of RADA16-I solution and RADA16-I-EM colloidal suspension

Took appropriate amount of RADA16-I powder, added sterilized ddH_2_O, and ultrasonically made it completely dissolve to obtain RDA16-I solution. The final concentration of RADA16-I used in the experiment was 5 mg/mL; took appropriate amount of EM powder and added it to 5 mg/mL RADA16-I solution, magnetically stirred for 48 h to obtain RADA16-I-EM colloidal suspension.

### 2.3. Cell lines and cell culture

LLC mouse lung cancer cell and 4T1 mouse breast cancer cell were purchased from Shanghai Fuheng Biotechnology Co., Ltd. (Shanghai, China). MLE12 normal mouse lung cells and HC11 normal mouse breast cells were purchased from Nanjing Saihongrui Biotechnology Co., Ltd. (Nanjing, China). These cells were cultured in Dulbecco’s modified Eagle medium (DMEM, Gibco, Carlsbad, CA) supplemented with 10% fetal bovine serum (FBS, Gibco, Carlsbad, CA), 100 U/mL penicillin, and 100 μg/mL streptomycin at 37 °C in a humidified atmosphere with 5% CO_2_.

### 2.4. MTT assay

LLC, 4T1, MLE12, and HC11 cells in logarithmic growth phase were collected by trypsinization, and the complete culture medium was re-suspended into single cell suspension. The cells were inoculated in 96-well plate (4000 cells per well) and incubated overnight. Then incubated with RADA16-I solution (0.5, 1.0, 2.0, 4.0, and 6.0 mg/mL) for 24 h, 48 h, and 72 h to evaluate the cell cytotoxicity. In the cell proliferation inhibition experiment, the cells were treated by free EM (EM water suspension (EMS), at the concentrations of EM as 60, 80, 100, 120, 140, 160, 180, and 200 μM) and RADA16-I-EM *in situ* hydrogels (in which the concentration of RADA16-I was 5.0 mg/mL, and concentration of EM were 60, 80, 100, 120, 140, 160, 180, and 200 μM) and cultured for 24 h and 72 h. Ten microliters MTT (5 mg/mL, Sigma, St. Louis, MO) was added into each well. After incubation at 37 °C for 4 h, the supernatant was removed and DMSO (Sigma, St. Louis, MO) was added into each well. A microplate reader (Bio-Rad Laboratories, Hercules, CA) was used to evaluate the cells viability at a wavelength of 490 nm.

$$\text{Cell viability}=\frac{A1-A0}{A2-A0}\times 100\%$$

where *A*1 refers to the absorbance of cells treated with drugs at 490 nm; *A*2 stands for the absorbance of cells in non-treated group and *A*0 is the absorbance of cells in non-cell group.

### 2.5. Colony formation assay

LLC cells were incubated with free EM and RADA16-I-EM *in situ* hydrogels (the final concentration of EM was 120, 160, and 200 μM) for 24 hours. The cells were collected by trypsinization. The drug treated and control cells were placed in a six-well plates at a density of 800 cells/well and maintained in medium containing 10% FBS. The culture medium was replaced every three days during colony growth. After 10 days, the cell colonies were washed with ice-cold PBS, fixed with pre-cooled methanol for 10 minutes, and stained with crystal violet for 15 minutes. Colonies were examined and calculated.

### 2.6. Cellular uptake assay

Cellular uptake of EM (self-green fluorescence) in RADA16-I-EM *in situ* hydrogels was evaluated in LLC cells. The cells were inoculated in a six-well plate at 37 °C for 24 h, then free EM and RADA16-I-EM *in situ* hydrogels (the final concentration of EM was 120, 160, and 200 μM) were added. After incubation in 37 °C for 24 h, the cells were washed with cold PBS for three times, digested with trypsinization, suspended in 300 μL of PBS, and analyzed by flow cytometry (Becton-Dickinson, San Jose, CA). For qualitative analysis, the cells were seeded on a confocal dish and cultured for 24 h, then incubated with free EM and RADA16-I-EM *in situ* hydrogels (the final concentration of EM was 120, 160, and 200 μM) for 24 h, then washed with cold PBS for three times and fixed with 4% paraformaldehyde for 30 min, and stained with DAPI for 20 min. The cells were then captured by confocal laser scanning microscope (Leica, Wetzlar, Germany).

### 2.7. Transwell migration/invasion assays

After incubating LLC cells with free EM and RADA16-I-EM *in situ* hydrogels (final concentration of EM was 120, 160, and 200 μM) for 24 h, the cells were collected by trypsinization for migration and invasion analysis. In the Transwell cell culture plate, 1 × 10^5^ cells were added to the upper chamber of the device at a serum-free concentration of 200 μL, and the lower chamber was filled with 700 μL culture medium with 20% FBS. After 15 h of incubation at 37 °C, the filters were fixed in methanol for 15 min, stained with crystal violet for 15 min and then the non-migration cells were removed from the upper surface of the membrane with a cotton swab. Nine random microscopic fields (×200) were counted per well, and calculate the average value. For the Transwell invasion experiment, the cell membrane of the upper chamber was pre-coated with 100 μL of 2.5 mg/mL Matrigel (BD Biosciences, Bedford, MA).

### 2.8. Cell apoptosis analysis

Annexin V-Alexa Fluor 647/PI dual staining was used to analyze apoptosis. LLC cells were inoculated in a six-well plate. After 24 hours of culture, the cells were treated with RADA16-I-EM *in situ* hydrogels and free drug EM (in which the final concentration of RADA16-I was 5.0 mg/mL, and EM were 120, 160, and 200 μM) for 24 h. The cells were collected, washed with cold PBS for three times, suspended in 300 μL binding buffer and stained with Annexin V-Alexa Fluor 647/PI. Finally, the cells were analyzed by flow cytometry (Becton-Dickinson, San Jose, CA).

### 2.9. Cell cycle analysis

The proportion of cells in the G0/G1, S, and G2/M phases was detected by flow cytometry. Briefly, LLC cells were incubated with free EM and RADA16-I-EM *in situ* hydrogels with EM concentration of 120, 160, and 200 μM for 24 h, then the cells were harvested by trypsinization and then fixed overnight with 75% ethanol at −20 °C. The fixed cells were centrifuged at 1000 rpm for five minutes and re-suspended in 300 μL staining buffer before detection, 10 μL propidium iodide (PI, 50 μg/mL) was added, and the suspension was incubated at 37 °C for 30 minutes in the dark at room temperature. Finally, the cells were detected by flow cytometry (Becton-Dickinson, San Jose, CA).

### 2.10. *In vivo* anti-lung cancer efficacy

Male C57 mice were purchased from Speyford Biotechnology Co., Ltd. (Beijing, China). The mice were kept in pathogen-free conditions. The mice were injected subcutaneously into the right flanks with 1 × 10^6^ cells/mL (100 μL) of LLC lung cancer cells. Ten days after inoculation, and the cancer volumes were calculated using the equation, *V* = 0.5 × length × width^2^. After the cancer volume reached 150–250 mm^3^, LLC lung cancer-bearing C57 mice were randomly divided into four groups (eight mice per group) with these treatments: PBS + 20% PEG400 (control), RADA16-I, free EM, and RADA16-I-EM *in situ* hydrogels. Fifty microliters of RADA16-I (5.0 mg/mL), free EM and RADA16-I-EM *in situ* hydrogels at an EM dose of 10 mg/kg every two days were injected into the cancers of mice *in situ*. The body weight and tumor size of mice were measured every two days after treatment. After treatment for 12 days, the animals were humanely sacrificed, and the main organs (heart, liver, spleen, lung, and kidneys) were collected and fixed with 10% paraformaldehyde for 48 h for histopathological examination to evaluate the adverse reactions. The cancer tissues were collected for HE staining, terminal deoxynucleotidyl transferase dUTP nick-end labeling (TUNEL), and Ki-67 antibody staining to detect the subcutaneous growth of the cancer. All animal care and experiments were conducted in accordance with National and Institutional Policies on Animal Health and Well-Being. The protocol was approved by the Institutional Animal Care and Use Committee of Zunyi Medical University (approval number: ZMUER2014-2-069).

### 2.11. Statistical analysis

All data were obtained from at least three independent experiments and all statistical analyses were performed using SPSS18.0 statistical software (SPSS Inc., Chicago, IL) or GraphPad Prism 5.0 software (GraphPad). All the obtained data are expressed as mean ± standard deviation (SD). One-way analysis of variance (ANOVA) with the LSD-*t* or the Dunnett-*t*-test was used for multi-group comparison, and Student's *t*-test was used for comparison between two groups. Differences were considered statistically significant when the *p* value was less than .05.

## 3. Results and discussion

### 3.1. Toxicity of RADA16-I *in situ* hydrogels

**Figure 3.**
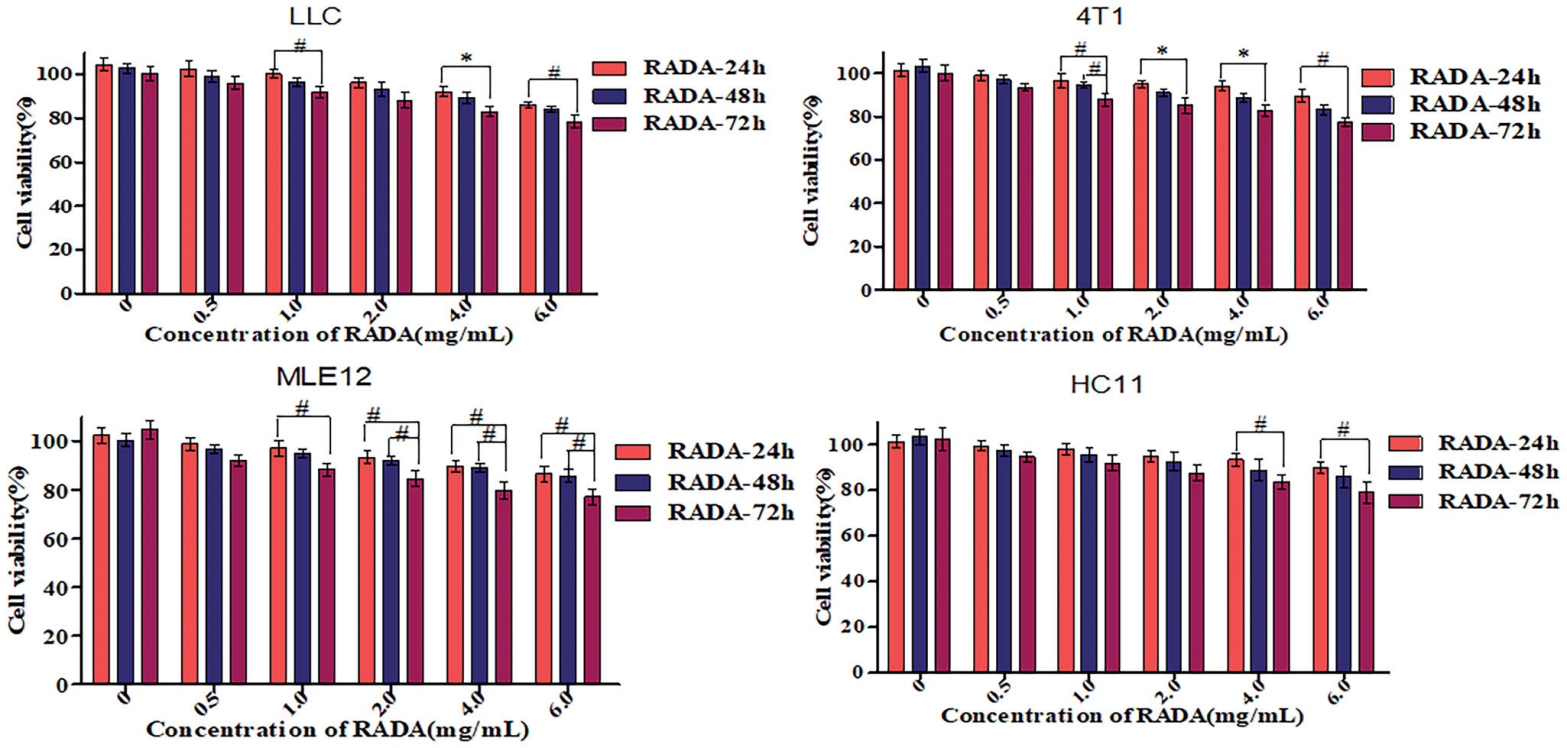
Viability of cells treated with various concentrations of RADA16-I *in situ* hydrogels for 24, 48, and 72 h. *^#^p*<.05, *p<.01, all values are expressed as the mean ± SD. RADA: self-assembling peptide RADA16-I; EM: emodin.

To assess the cytotoxicity of RADA16-I *in situ* hydrogels *in vitro*, the viability of four cell lines (LLC, 4T1, MLE12, and HC11 cells) incubated with the RADA16-I at the concentration range of 0.5–6.0 mg/mL, was evaluated using MTT assay in the time and concentration dependent manner. As shown in Figure 3, in four cell lines, at the same time, the cytotoxicity increased slowly with the increase of the concentration of RADA16-I solution; furthermore, at the same concentration, the cytotoxicity also increased slightly with the prolonged of the time. However, the final survival rate of cells is close to or greater than 85% in the range of 0.5–6.0 mg/mL, indicating that the peptide RADA16-I has low toxicity to cells and self-assembling peptide RADA16-I has good cell compatibility as a new drug carrier.

### 3.2. Cytotoxicity of RADA16-I-EM *in situ* hydrogel

**Figure 4.**
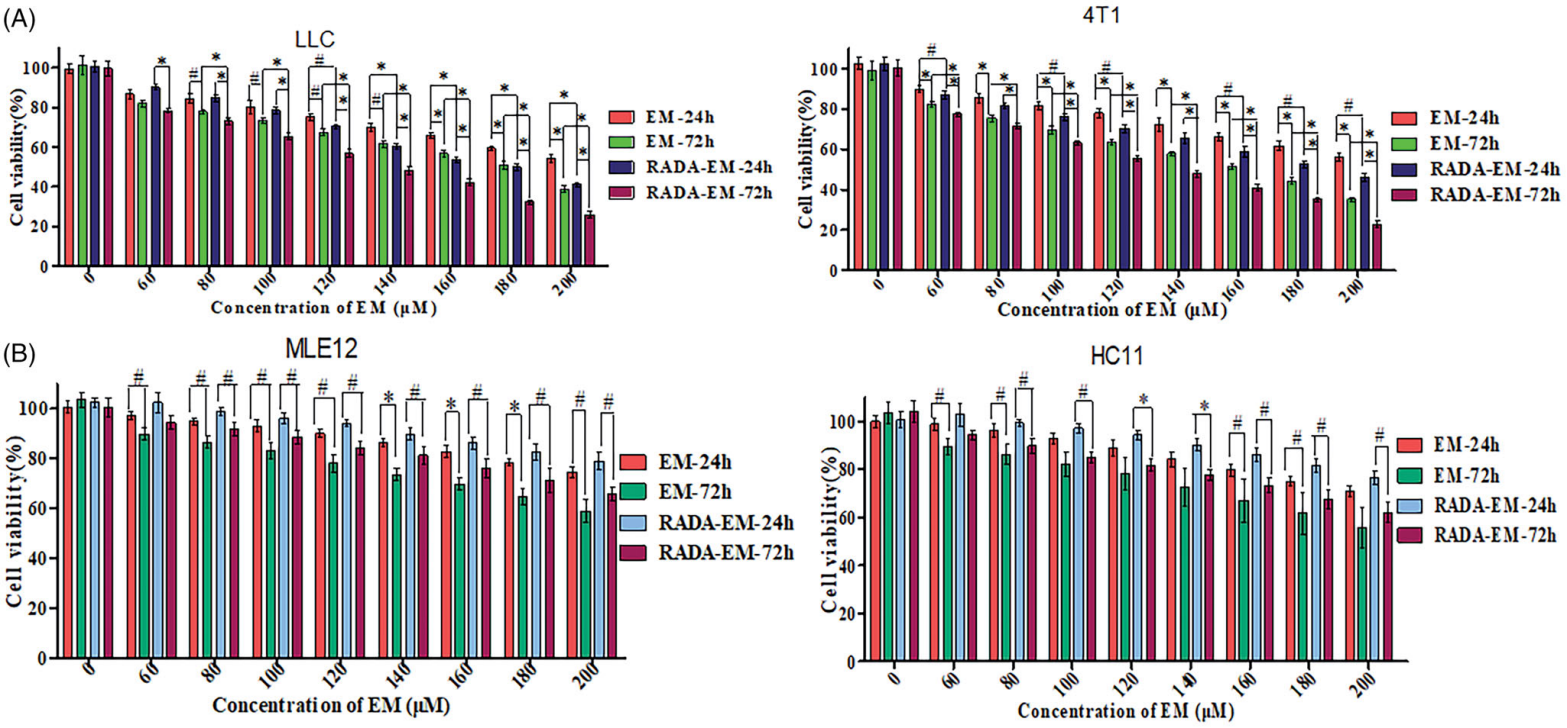
MTT viability assays of cells with different treatments of free EM and RADA16-I-EM *in situ* hydrogels for 24 and 72 h. [RADA16-I] = 5 mg/mL, *^#^p*<.05, *p<.01, all values are expressed as the mean ± SD. RADA: self-assembling peptide RADA16-I; EM: emodin.

The cytotoxicity of EM in different formulations against the cancer cells or normal cells was investigated via an MTT assay. The obtained results are shown in Figure 4(A), the inhibitory effect of different EM formulations on cell proliferation was concentration-dependent and time-dependent. After incubation with free EM and RADA16-I-EM *in situ* hydrogels for 24 h and 72 h, much higher proliferation inhibition rate was observed in cells treated with RADA16-I-EM *in situ* hydrogels than free EM, which indicated that RADA16-I-EM *in situ* hydrogels could promote the release of EM. It can be concluded that RADA16-I can improve the solubility and increase the dissolution of EM to improve the inhibitory effect of EM on the proliferation of cancer cells. Interestingly, as shown in Figure 4(B), with the increase of drug EM concentration and incubation time, both RADA16-I-EM *in situ* hydrogels and free EM had certain cytotoxicity to normal cells, but the cytotoxicity of RADA16-I-EM *in situ* hydrogels was lower than free EM. This may be due to the slow release of EM into cells by RADA16-I-EM *in situ* hydrogels, which did not immediately reach the minimum toxic concentration of the drug, so its toxicity to normal cells was low.

### 3.3. Rada16-I-EM *in situ* hydrogels decreases cell proliferation in LLC cancer cells

**Figure 5.**
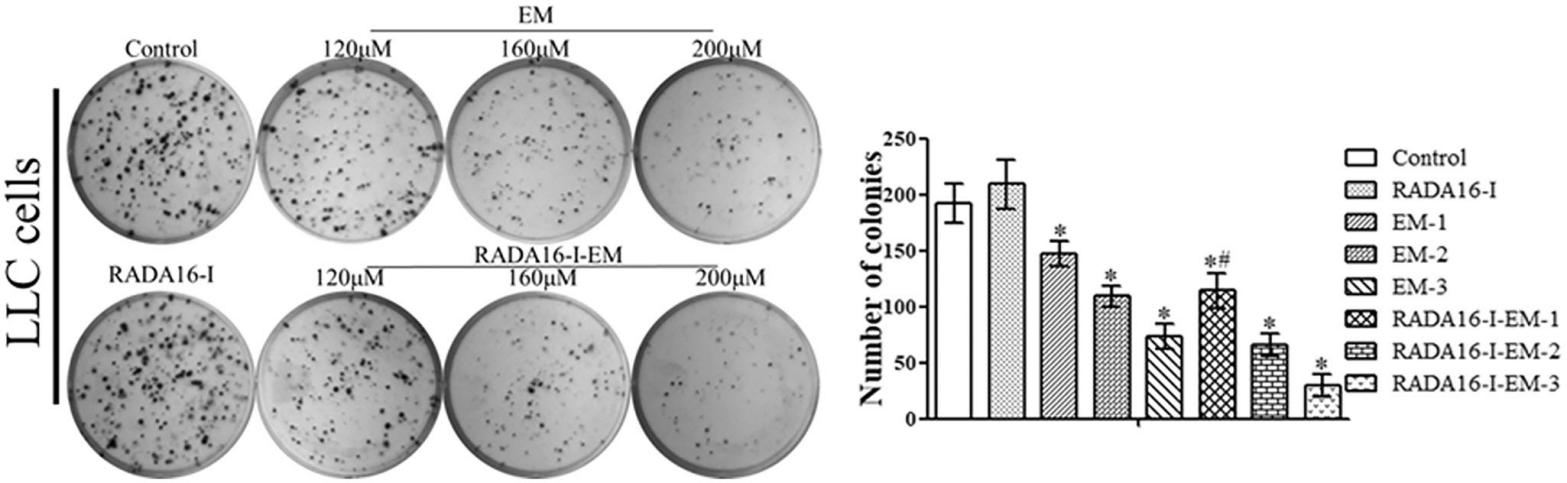
Images of colony formation assay in free EM and RADA16-I-EM *in situ* hydrogels treated LLC cells. *p< .01, *^#^p*< .05 vs. EM or control. All values are expressed as the mean ± SD.

Cell clone formation assay is an important index to evaluate cell population dependence. Only cancer cells or cells with the tendency of malignant lesions can form colonies, and the number of colonies formed by a single cell clone is greater than or equal to 30 cells as the standard, which can reflect the clone formation ability of a single cell and the ability of malignant transformation (Mao, 2014). Therefore, in order to further evaluate the proliferation of LLC cells treated with RADA16-I-EM *in situ* hydrogels, colony formation tests were carried out to examine the long-term effects caused by drug-loaded *in situ* hydrogels. As shown in the staining plates, RADA16-I *in situ* hydrogels had no significant effect on cell proliferation compared with control, while RADA16-I-EM *in situ* hydrogels remarkably reduced the quantity and colony size of LLC cells compared with free EM (*p*<.05, Figure 5).

### 3.4. Cellular uptake of EM in RADA16-I-EM *in situ* hydrogels

**Figure 6.**
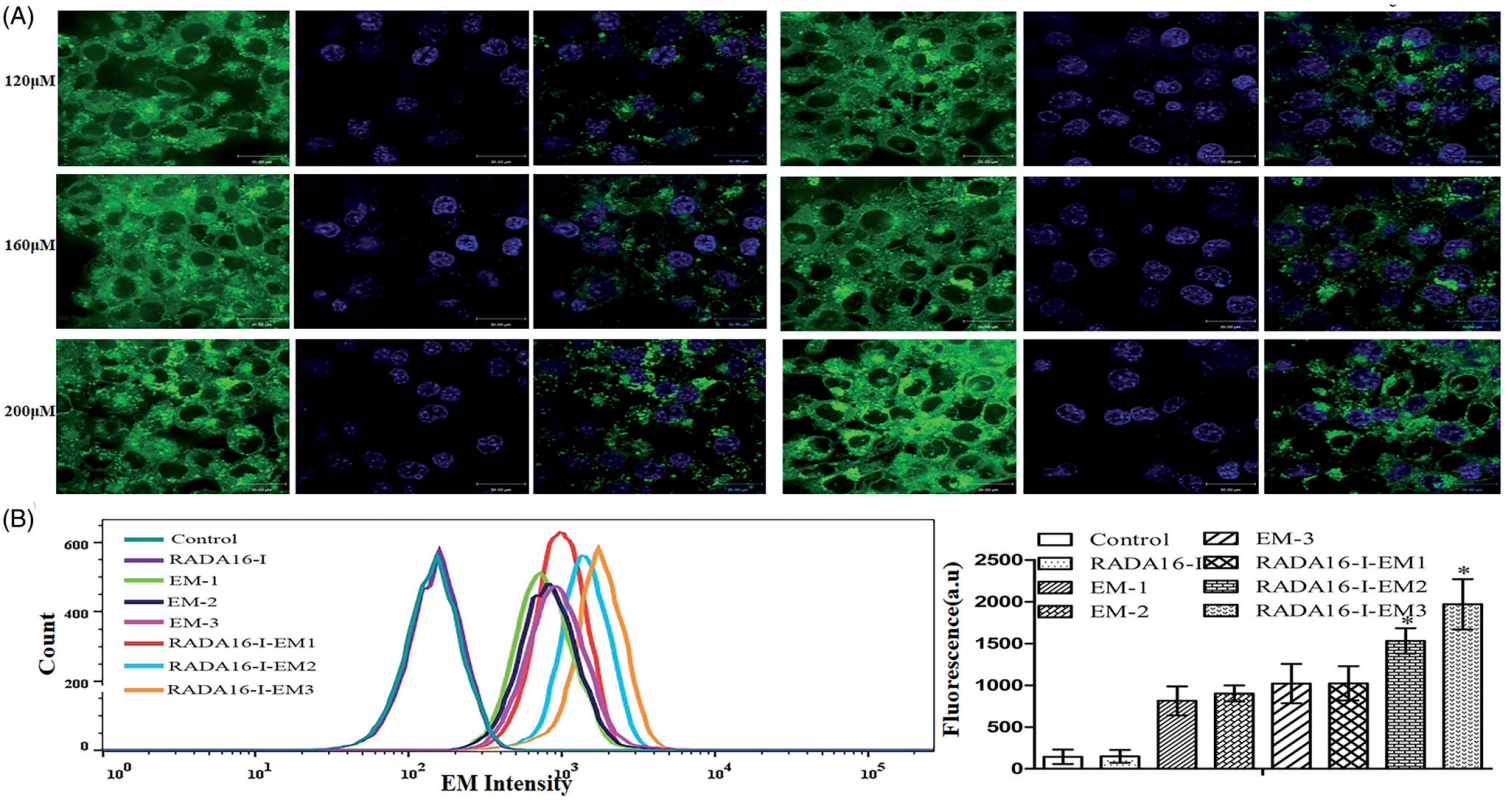
Cellular uptake of EM in different formulations. (A) Cellular uptake of EM with or without RADA16-I. (B) Cellular uptake of EM in different formulations via flow cytometry. 1, 2, and 3 represent EM concentrations of 120, 160, and 200 μM, respectively, [RADA16-I] = 5 mg/mL, *p< .01 vs. EM.

In order to achieve efficient cytotoxicity, the *in situ* hydrogel loaded with EM should be internalized into the cell to ensure the intracellular EM concentration. As shown in Figure 6, after 24 h incubation, RADA16-I-EM *in situ* hydrogels-treated cells exhibited relatively high EM intensity in the cytoplasm, while the EM uptake was significantly lower in the cells incubated with free EM. Later, the quantitatively cellular uptake of EM in different formulations was investigated via flow cytometry. After 24 h of incubation, RADA16-I-EM *in situ* hydrogels exhibited a higher cellular uptake than free EM (*p*<.01), suggesting that EM loading into RADA16-I could help to enhance the intracellular drug concentration, and the conclusion was the same as the confocal result. It is demonstrated that RADA16-I-EM *in situ* hydrogels can be effectively absorbed by cells and EM can be transferred to the cytoplasm.

### 3.5. RADA16-I-EM *in situ* hydrogels inhibits the migration and invasion of LLC cells

**Figure 7.**
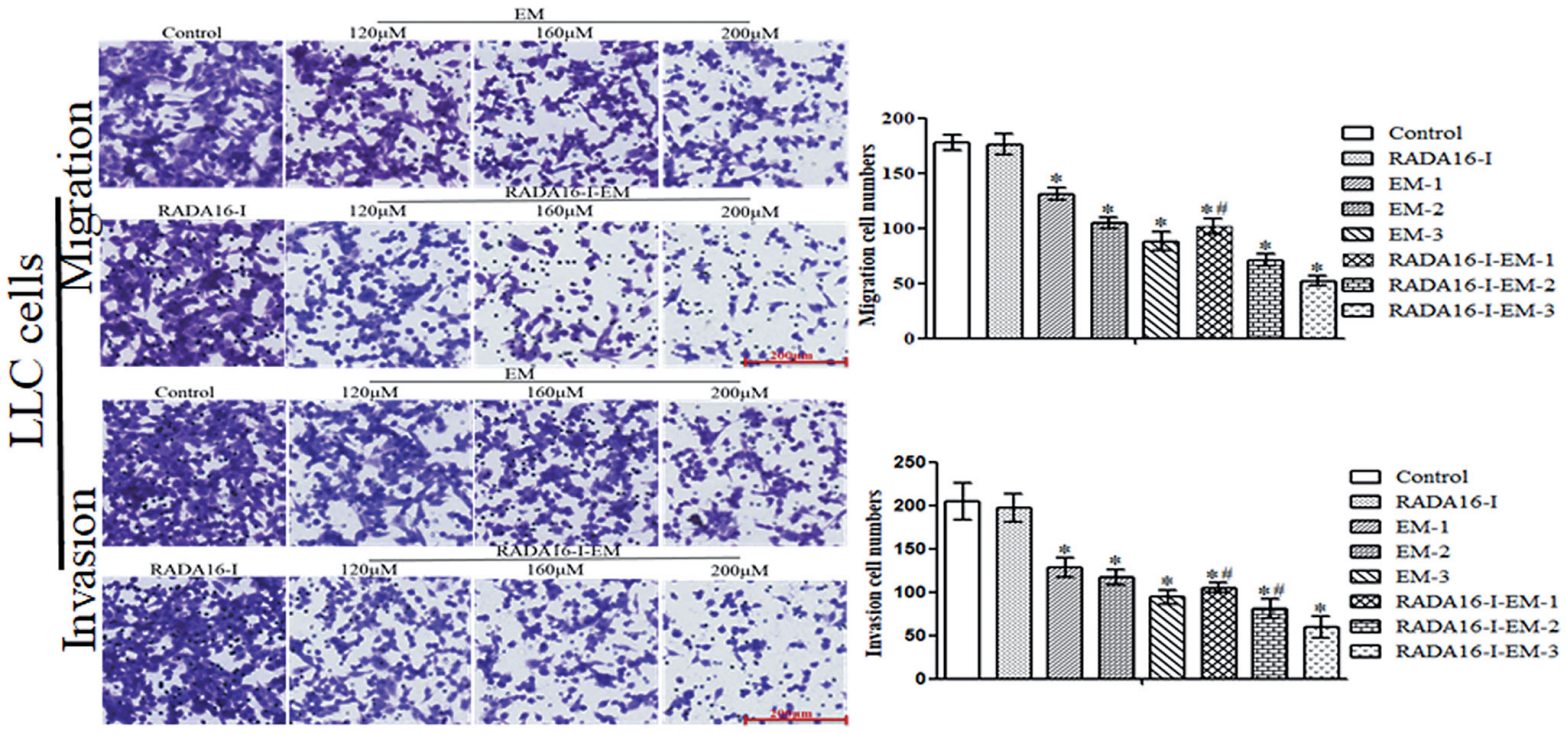
Representative images of the migration and invasion assays in free EM and RADA16-I-EM *in situ* hydrogels treated LLC cells (left panel). The cell migration and invasion rate at 24 h is shown in right panel. Scale bar, 200 µm. *p< .01, *^#^p*< .05 vs. EM or control. All values are expressed as mean ± SD.

The migration and invasion ability of LLC cells were then examined using Transwell assays. In terms of the migration and invasion ability after 15 h as shown by the test assays, we found that LLC cells treated with *in situ* hydrogels without drug RADA16-I had no statistically significant difference compared with control cells. But the migration and invasion ability of the cells treated with free EM or RADA16-I-EM *in situ* hydrogels was significantly decreased, and after RADA16-I-EM *in situ* hydrogels treatment, the migration and invasion ability of LLC cells was significantly lower than that of cells treated with free EM (Figure 7).

### 3.6. Effect of RADA16-I-EM *in situ* hydrogel on cell apoptosis of LLC cells

**Figure 8.**
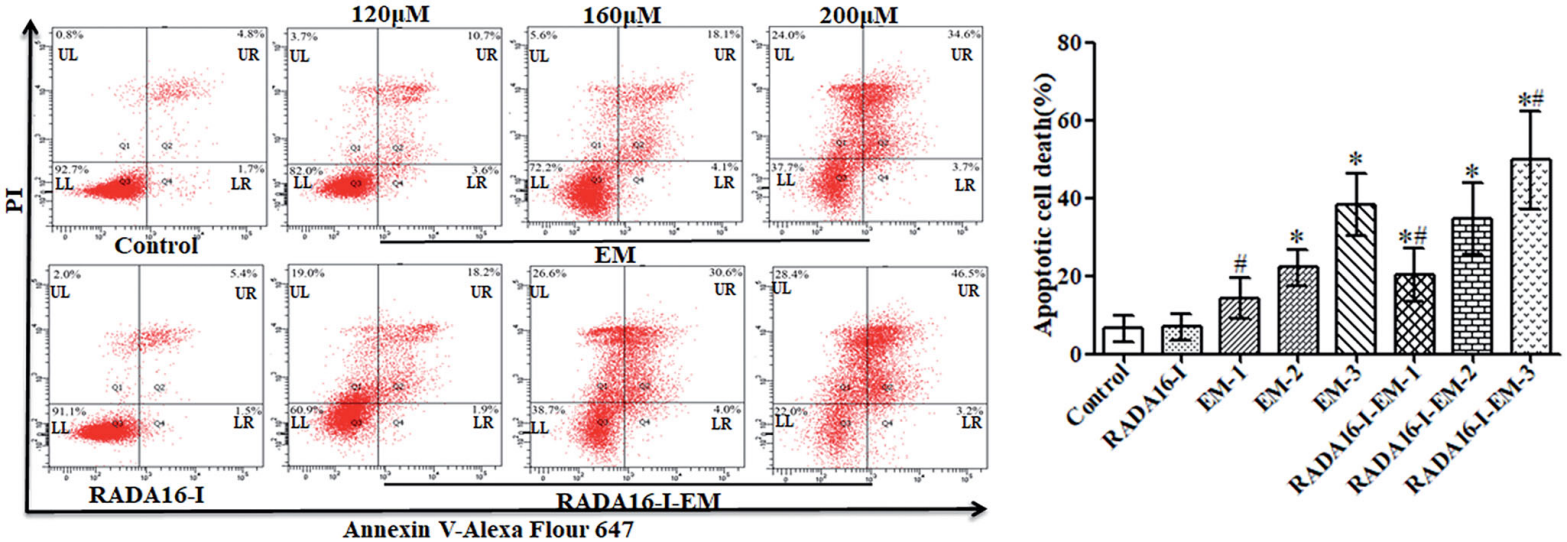
Representative images of Annexin V/PI analysis and apoptosis percentage of LLC cells with various EM formulations. 1, 2, and 3 represent EM concentrations of 120, 160, and 200 μM, respectively, [RADA16-I] = 5 mg/mL, *^#^p*< .05, *p< .01 vs. EM or control group.

In order to further evaluate the effect of RADA16-I-EM *in situ* hydrogels on the apoptosis of LLC cells, the apoptosis of LLC cells was detected by flow cytometry. As shown in Figure 8, RADA16-I *in situ* hydrogels without drug loading had no significant effect on LLC cells, while free EM and RADA16-I-EM *in situ* hydrogels could induce cell apoptosis and necrosis, among which RADA16-I-EM *in situ* hydrogels induced LLC cell apoptosis in the early and late stages with the highest rate of 49.7%, compared with 38.3% induced by free EM. These results indicated that RADA16-I could significantly enhance EM induced apoptosis of LLC cells, which might be due to the fact that RADA16-I could stabilize the hydrophobic drug EM, so to increase its solubility, and successfully release EM into cancer cells, thus improving cancer cells uptake efficiency and promoting cancer cells apoptosis.

### 3.7. Effect of RADA16-I-EM *in situ* hydrogels on cell cycle progression of LLC cells

**Figure 9.**
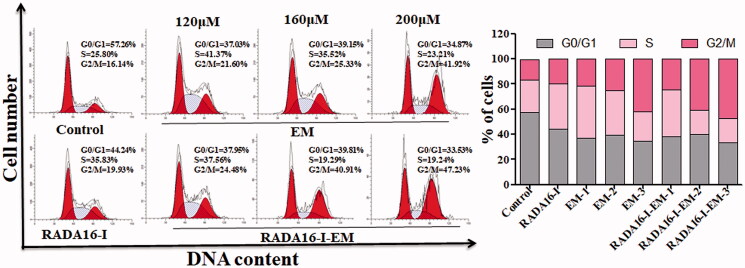
Flow cytometry analysis of cell cycle distribution in LLC cells treated with EM or RADA16-I-EM *in situ* hydrogels for 24 h. 1, 2, and 3 represent EM concentrations of 120, 160, and 200 μM, respectively, [RADA16-I] = 5 mg/mL.

As cell cycle progression plays an important role in determining cell growth, we then aimed to explore whether the insoluble drug EM packaged in RADA16-I could promote the cell cycle progression of LLC cells after forming hydrogels *in situ*. The results of the cell cycle experiment are shown in Figure 9. We found that the cell cycle distribution was not significantly changed in LLC cells after 24 h of incubation with RADA16-I *in situ* hydrogels, indicating that RADA16-I did not affect the cell cycle progression. However, a decrease in G0/G1 and S phase and an increase in G2/M phase were observed in LLC cells treated with free EM and RADA16-I-EM *in situ* hydrogels compared with control cells. The RADA16-I-EM *in situ* hydrogels significantly arrest LLC cells at the G2/M phase compared with the free EM group, indicating that RADA16-I-EM has a strong ability to inhibit the division and proliferation of cancer cell.

### 3.8. *In vivo* cancer suppression evaluation and systemic toxicity evaluation

**Figure 10.**
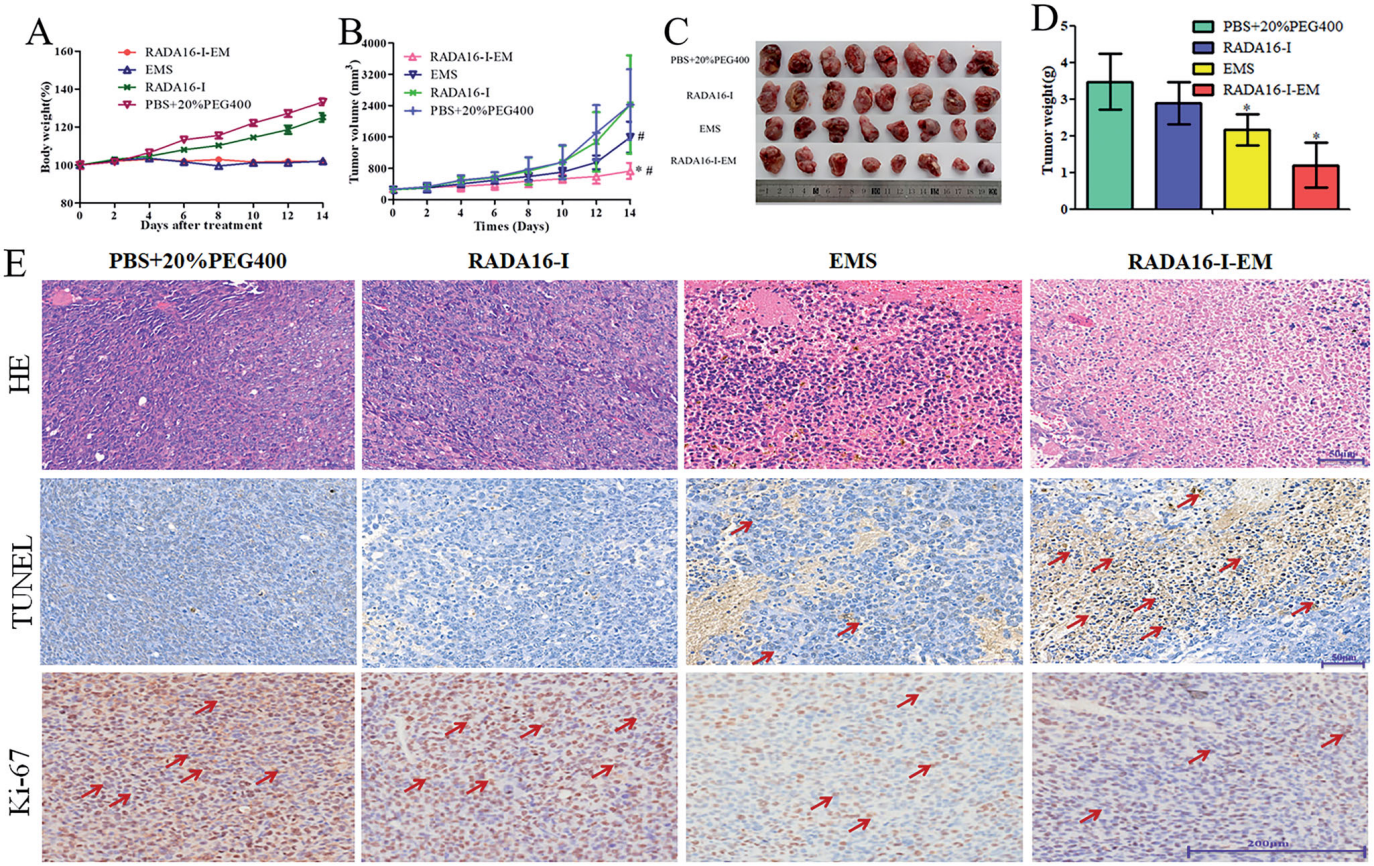
*In vivo* anticancer efficacy evaluation. (A) Body weights of mice after different treatments. (B) Cancer growth curves of different groups of cancer-bearing mice after various treatments indicated for 12 days. (C) Pictures of the harvested cancers. (D) Cancer weight of different groups. *p< .01 vs. PBS; *^#^p*< .05 vs. EMS or PBS. Data were presented as mean ± SD, *n* = 8. (E) H&E, TUNEL, and Ki-67 staining in cancer sections collected from differently treated groups of mice. ‘↗’ represents cancer cells apoptosis and proliferation, respectively.

**Figure 11.**
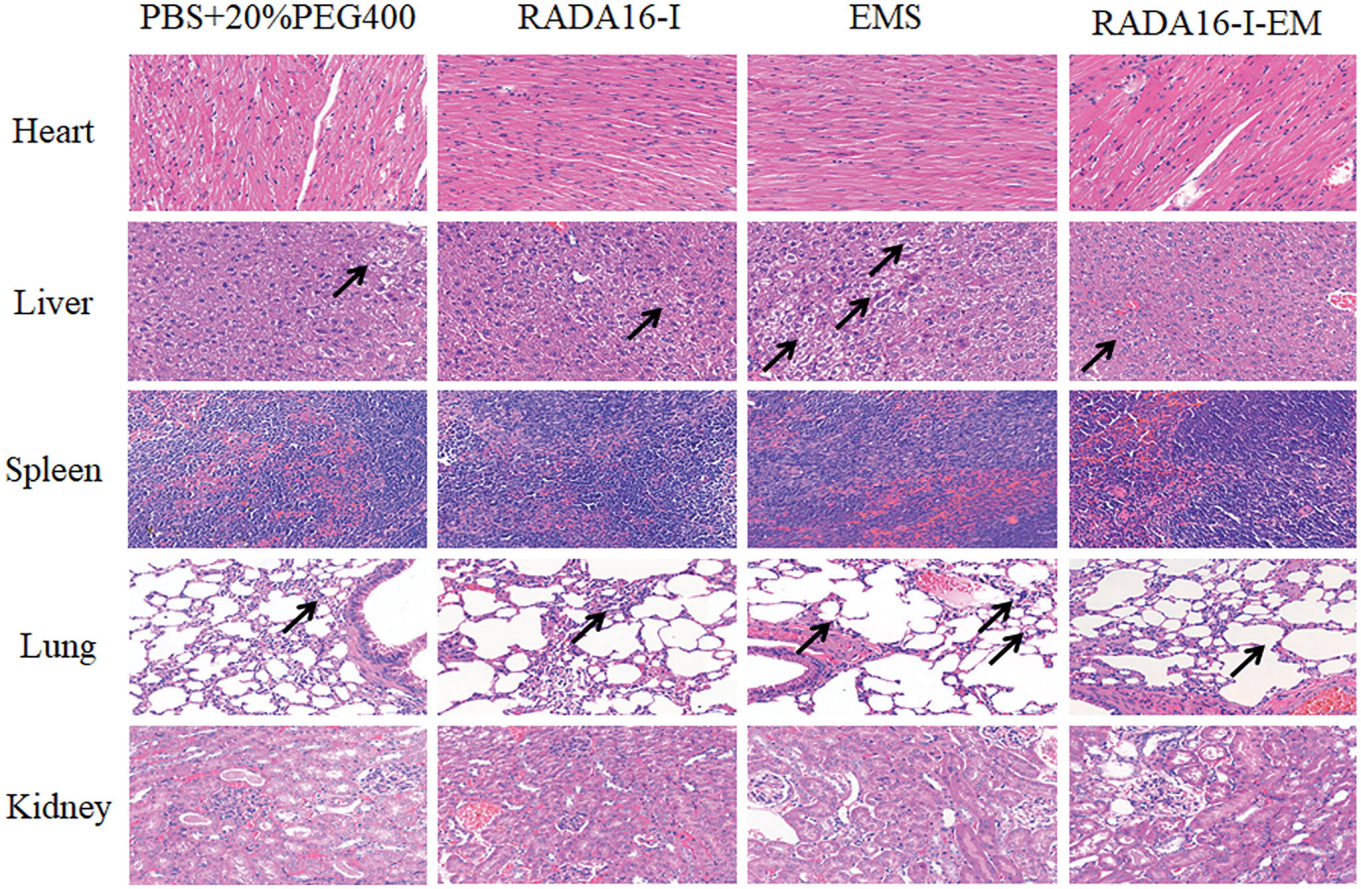
H&E staining of major organs from differently treated groups at day 12 (scale bar: 50 μm). ‘↗’ represents the toxic site of the organs tissues during treatment.

EM in different formulations was injected respectively into LLC cancer-bearing mice and the anticancer effect of RADA16-I-EM *in situ* hydrogels was monitored by measuring the cancer volume of each group. Meanwhile, the systemic toxicity of mice was determined by measuring the body weight and the injury of main organs (heart, liver, spleen, lung, and kidneys). The results are shown in Figure 10(A). There was no significant decrease in body weight in each group during treatment. LLC cancers grew extremely rapidly in the control and RADA16-I groups, whereas EM in different formulations had obvious inhibitory effect on cancer growth, especially in the RADA16-I-EM *in situ* hydrogels group (Figure 10(B)). Moreover, RADA16-I-EM *in situ* hydrogels exhibited a better anticancer effect than free EM due to the efficient cellular uptake *in vivo*. As shown in Figure 10(C), harvested cancers of the RADA16-I-EM *in situ* hydrogels group were the smallest. RADA16-I-EM *in situ* hydrogels-treated mice had the highest degree of cancer inhibition with significantly decreased cancer weights compared with other groups (Figure 10(D), *p*< .01). The cancer slices were then stained. The RADA16-I-EM *in situ* hydrogels group had largest necrotic areas and the most significant apoptosis in cancer tissue. It also has the lowest number of Ki-67-positive cells compared with other groups, as evidenced by HE, TUNEL, and Ki-67 (Figure 10(E)). EM water suspension, without selected accumulation in cancer tissues, exhibited a poor anticancer effect. As shown in Figure 11, during the treatment period, hepatocytes in all groups showed varying degrees of vacuolar degeneration and inflammatory cell infiltration, of which in EMS group hepatocyte vacuolar degeneration was the severest, followed by RADA16-I-EM *in situ* hydrogel group, and mild in PBS and RADA16-I groups. At the same time, there was inflammatory cell infiltration in the pulmonary interstitial in all groups, and the pulmonary interstitial was widened in EMS and RADA16-I-EM groups, which may be due to the greater effect of LLC cancer metastasis or drug toxicity on the liver and lung of animals. But in a word, compared with control group, the toxic effect of RADA16-I-EM *in situ* hydrogels on organs was lower than free EM. The above experimental results showed that RADA16-I could improve the solubility of insoluble drug EM and increase the local concentration of EM in cancer tissue, which could not only enhance the therapeutic effect of drug on cancer, but also significantly reduce the toxicity of drug to normal tissue.

## 4. Conclusions

In this study, based on the poor solubility of the hydrophobic drug EM in use, we chose the nanomaterial of self-assembling peptide RADA16-I as a carrier to prepare an EM-loaded RADA16 colloidal suspension (RADA16-I-EM). The solution could immediately form hydrogels *in situ* under physiological conditions and could be used as a preparation for local administration to improve the low solubility of EM. The drug was accumulated in cancer tissue through the sustained release effect of *in situ* hydrogels, and then absorbed by cells, thus enhancing the anti-cancer effect. *In vitro* experiments showed that RADA16-I-EM *in situ* hydrogels had the best anti-proliferation activity compared with control and free drugs, and *in vivo* studies in LLC lung cancer-bearing mice showed that the drug-loaded *in situ* hydrogels could effectively inhibit cancer growth with good biosafety. In conclusion, RADA16-I-EM *in situ* hydrogels had the behavior of slow-release drugs, could provide a promising strategy for anticancer drugs *in vivo* to control drug release.

## References

[CIT0001] Alisi A, Pastore A, Ceccarelli S, et al. (2012). Emodin prevents intrahepatic fat accumulation, inflammation and redox status imbalance during diet-induced hepatosteatosis in rats. Int J Mol Sci 13:2276–89. 22408453 10.3390/ijms13022276 PMC3292022

[CIT0002] Almeida H, Amaral MH, Lobao P, et al. (2014). *In situ* gelling systems: a strategy to improve the bioavailability of ophthalmic pharmaceutical formulations. Drug Discov Today 19:400–12. 24120893 10.1016/j.drudis.2013.10.001

[CIT0003] Ashwanikumar N, Kumar NA, Saneesh Babu PS, et al. (2016). Self-assembling peptide nanofibers containing phenylalanine for the controlled release of 5-fluorouracil. Int J Nanomedicine 11:5583–94. 27822037 10.2147/IJN.S104707 PMC5087806

[CIT0004] Bykov VJN, Eriksson SE, Bianchi J, et al. (2018). Targeting mutant p53 for efficient cancer therapy. Nat Rev Cancer 18:89–102. 29242642 10.1038/nrc.2017.109

[CIT0005] Chu K, Chen L, Xu W, et al. (2013). Preparation of a paeonol-containing temperature-sensitive *in situ* gel and its preliminary efficacy on allergic rhinitis. Int J Mol Sci 14:6499–515. 23525047 10.3390/ijms14036499 PMC3634513

[CIT0006] Cong Z, Zhang L, Ma SQ, et al. (2020). Size-transformable hyaluronan stacked self-assembling peptide nanoparticles for improved transcellular tumor penetration and photo-chemo combination therapy. ACS Nano 14:1958–70. 32023048 10.1021/acsnano.9b08434

[CIT0007] Cullen JK, Simmons JL, Parsons PG, et al. (2020). Topical treatments for skin cancer. Adv Drug Deliv Rev 153:54–64. 31705912 10.1016/j.addr.2019.11.002

[CIT0008] Debele TA, Lee KY, Hsu NY, et al. (2017). A pH sensitive polymeric micelle for co-delivery of doxorubicin and α-TOS for colon cancer therapy. J Mater Chem B 5:5870–80. 32264220 10.1039/c7tb01031a

[CIT0009] Ding J, Zhang J, Li J, et al. (2019). Electrospun polymer biomaterial. Prog Polym Sci 90:1–34.

[CIT0010] Eskandari S, Guerin T, Toth I, et al. (2017). Recent advances in self-assembled peptides: implications for targeted drug delivery and vaccine engineering. Adv Drug Deliv Rev 110–111:169–87. 10.1016/j.addr.2016.06.013 27356149

[CIT0011] Feng X, Li J, Zhang X, et al. (2019). Electrospun polymer micro/nanofibers as pharmaceutical repositories for healthcare. J Control Release 302:19–41. 30922946 10.1016/j.jconrel.2019.03.020

[CIT0012] Fu ZY, Han JX, Huang HY. (2007). Effects of emodin on gene expression profile in small cell lung cancer NCI-H446 cells. Chin Med J 120:1710–5. 17935676

[CIT0013] He C, Ma H, Cheng Y, et al. (2015). PLK1shRNA and doxorubicin co-loaded thermosensitive PLGA-PEG-PLGA hydrogels for localized and combined treatment of human osteosarcoma. J Control Release 213:e18. 10.1016/j.jconrel.2015.05.026 27005126

[CIT0014] Hu X, Wang Y, Zhang L, et al. (2017). Redox/pH dual stimuli-responsive degradable Salecan-g-SS-poly(IA-co-HEMA) hydrogel for release of doxorubicin. Carbohydr Polym 155:242–51. 27702509 10.1016/j.carbpol.2016.08.077

[CIT0015] Khaliq NU, Oh KS, Sandra FC, et al. (2017). Assembly of polymer micelles through the sol–gel transition for effective cancer therapy. J Control Release 255:258–69. 28456679 10.1016/j.jconrel.2017.04.039

[CIT0016] Lee YS, Kang OH, Choi JG, et al. (2010). Synergistic effect of emodin in combination with ampicillin or oxacillin against methicillin-resistant *Staphylococcus aureus*. Pharm Biol 48:1285–90. 20925591 10.3109/13880201003770150

[CIT0017] Li WY, Chan RY, Yu PH, et al. (2013). Emodin induces cytotoxic effect in human breast carcinoma MCF-7 cell through modulating the expression of apoptosis-related genes. Pharm Biol 51:1175–81. 23763280 10.3109/13880209.2013.782322

[CIT0018] Li S, Dong S, Xu W, et al. (2018). Antibacterial hydrogels. Adv Sci 5:1700527. 10.1002/advs.201700527 PMC5980143 29876202

[CIT0019] Lin G, Chen CK, Yin F, et al. (2017). Biodegradable nanoparticles as siRNA carriers for in vivo gene silencing and pancreatic cancer therapy. J Mater Chem B 5:3327–37. 32264398 10.1039/c6tb03116a

[CIT0020] Liu J, Zhang L, Yang Z, et al. (2011). Controlled release of paclitaxel from a self-assembling peptide hydrogel formed *in situ* and antitumor study in vitro. Int J Nanomedicine 6:2643–53. 10.2147/IJN.S24038 PMC3215155 22114478

[CIT0021] Lu Y, Yang JH, Li X, et al. (2011). Emodin, a naturally occurring anthraquinone derivative, suppresses IgE-mediated anaphylactic reaction and mast cell activation. Biochem Pharmacol 82:1700–8. 21907188 10.1016/j.bcp.2011.08.022

[CIT0022] Mahvi DA, Liu R, Grinstaff MW, et al. (2018). Local cancer recurrence: the realities, challenges, and opportunities for new therapies. CA Cancer J Clin 68:488–505. 30328620 10.3322/caac.21498 PMC6239861

[CIT0023] Mao Z. (2014). Minor clone may drive cancer growth. Cancer Discov 4:1109–10. 10.1158/2159-8290.CD-NB2014-126 25274666

[CIT0024] Meng C, Wei W, Wang Y, et al. (2019). Study of the interaction between self-assembling peptide and mangiferin and in vitro release of mangiferin from in situ hydrogel. Int J Nanomedicine 14:7447–60. 31686816 10.2147/IJN.S208267 PMC6751768

[CIT0025] Milcovich G, Lettieri S, Antunes FE, et al. (2017). Recent advances in smart biotechnology: hydrogels and nanocarriers for tailored bioactive molecules depot. Adv Colloid Interface Sci 249:163–80. 28527520 10.1016/j.cis.2017.05.009

[CIT0026] Morsi N, Ghorab D, Refai H, et al. (2016). Ketoroloac tromethamine loaded nanodispersion incorporated into thermosensitive *in situ* gel for prolonged ocular delivery. Int J Pharm 506:57–67. 27091293 10.1016/j.ijpharm.2016.04.021

[CIT0027] Norouzi M, Nazari B, Miller DW. (2016). Injectable hydrogel-based drug delivery systems for local cancer therapy. Drug Discov Today 21:1835–49. 27423369 10.1016/j.drudis.2016.07.006

[CIT0028] Ostrom QT, Gittleman H, Liao P, et al. (2017). CBTRUS statistical report: primary brain and other central nervous system tumors diagnosed in the United States in 2010–2014. Neuro-Oncology 19:v1–v88. 29117289 10.1093/neuonc/nox158 PMC5693142

[CIT0029] Pan A, Wang Z, Chen B, et al. (2018). Localized co-delivery of collagenase and trastuzumab by thermosensitive hydrogels for enhanced antitumor efficacy in human breast xenograft. Drug Deliv 25:1495–503. 29943651 10.1080/10717544.2018.1474971 PMC6058501

[CIT0030] Park K. (2018). Enhanced bacterial cancer therapy with hydroxychloroquine liposomes. J Control Release 280:124. 29880282 10.1016/j.jconrel.2018.05.028

[CIT0031] Park B, Yoon W, Yun J, et al. (2019). Emodin-nicotinamide (1:2) cocrystal identified by thermal screening to improve emodin solubility. Int J Pharm 557:26–35. 30572078 10.1016/j.ijpharm.2018.12.027

[CIT0032] Qiu F, Chen Y, Tang C, et al. (2018). Amphiphilic peptides as novel nanomaterials: design, self-assembly and application. Int J Nanomedicine 13:5003–22. 30214203 10.2147/IJN.S166403 PMC6128269

[CIT0033] Qiu H, Guo H, Li D, et al. (2020). Intravesical hydrogels as drug reservoirs. Trends Biotechnol 38:579–83. 31926600 10.1016/j.tibtech.2019.12.012

[CIT0034] Saravanan S, Vimalraj S, Thanikaivelan P, et al. (2019). A review on injectable chitosan/beta glycerophosphate hydrogels for bone tissue regeneration. Int J Biol Macromol 121:38–54. 30291931 10.1016/j.ijbiomac.2018.10.014

[CIT0035] Shamsi F. (2016). Investigation of human cell response to covalently attached RADA16-I peptide on silicon surfaces. Colloids Surf B Biointerfaces 145:470–8. 27236098 10.1016/j.colsurfb.2016.05.030

[CIT0036] Taghavi L, Aramvash A, Seyedkarimi MS, et al. (2018). Evaluation of the hemocompatibility of RADA 16-I peptide. J Biomater Appl 32:1024–31. 29249197 10.1177/0885328217748861

[CIT0037] Tarvirdipour S, Schoenenberger CA, Benenson Y, et al. (2020). A self-assembling amphiphilic peptide nanoparticle for the efficient entrapment of DNA cargoes up to 100 nucleotides in length. Soft Matter 16:1678–91. 31967171 10.1039/c9sm01990a

[CIT0038] Tu Y, Wu Z, Tan B, et al. (2019). Emodin: its role in prostate cancer-associated inflammation (review). Oncol Rep 42:1259–71. 31524243 10.3892/or.2019.7264

[CIT0039] Wang W, Song H, Zhang J, et al. (2015). An injectable, thermosensitive and multicompartment hydrogel for simultaneous encapsulation and independent release of a drug cocktail as an effective combination therapy platform. J Control Release 203:57–66. 25683618 10.1016/j.jconrel.2015.02.015

[CIT0040] Wei L, Chen J, Zhao S, et al. (2017). Thermo-sensitive polypeptide hydrogel for locally sequential delivery of two-pronged antitumor drugs. Acta Biomater 58:44–53. 28576715 10.1016/j.actbio.2017.05.053

[CIT0041] Wei W, Li H, Yin C, et al. (2020). Research progress in the application of *in situ* hydrogel system in tumor treatment. Drug Deliv 27:460–8. 32166987 10.1080/10717544.2020.1739171 PMC7144265

[CIT0042] Wei W, Meng C, Wang Y, et al. (2019). The interaction between self-assembling peptides and emodin and the controlled release of emodin from in-situ hydrogel. Artif Cells Nanomed Biotechnol 47:3961–75. 31588802 10.1080/21691401.2019.1673768

[CIT0043] Wei W, Tang J, Li H, et al. (2021). Antitumor effects of self-assembling peptide-emodin *in situ* hydrogels *in vitro* and *in vivo*. Int J Nanomedicine 16:47–60. 33442249 10.2147/IJN.S282154 PMC7797320

[CIT0044] Wu D, Zhang S, Zhao Y, et al. (2018). The effects of motif net charge and amphiphilicity on the self-assembly of functionally designer RADA16-I peptides. Biomed Mater 13:035011. 29546848 10.1088/1748-605X/aab2fd

[CIT0045] Xiang J, Wu B, Zhou Z, et al. (2018). Synthesis and evaluation of a paclitaxel-binding polymeric micelle for efficient breast cancer therapy. Sci China Life Sci 61:436–47. 29572777 10.1007/s11427-017-9274-9

[CIT0046] Xiong L, Luo Q, Wang Y, et al. (2015). An injectable drug-loaded hydrogel based on a supramolecular polymeric prodrug. Chem Commun 51:14644–7. 10.1039/c5cc06025g 26290273

[CIT0047] Yin X, Gu Y, Qin L, et al. (2021). Injectable thermosensitive hydrogel-based drug delivery system for local cancer therapy. Colloids Surf B Biointerfaces 200:111581. 33524696 10.1016/j.colsurfb.2021.111581

[CIT0048] Zeng J, Shi D, Gu Y, et al. (2019). Injectable and near-infrared-responsive hydrogels encapsulating dopamine-stabilized gold nanorods with long photothermal activity controlled for tumor therapy. Biomacromolecules 20:3375–84. 31389691 10.1021/acs.biomac.9b00600

[CIT0049] Zhang Y, Yu J, Ren K, et al. (2019). Thermosensitive hydrogels as scaffolds for cartilage tissue engineering. Biomacromolecules 20:1478–92. 30843390 10.1021/acs.biomac.9b00043

[CIT0050] Zhu Y, Liang J, Gao C, et al. (2020). Multifunctional ginsenoside Rg3-based liposomes for glioma targeting therapy. J Control Release 330:1–55. 33359582 10.1016/j.jconrel.2020.12.036

